# EEG-Based Emotion Recognition by Convolutional Neural Network with Multi-Scale Kernels

**DOI:** 10.3390/s21155092

**Published:** 2021-07-27

**Authors:** Tran-Dac-Thinh Phan, Soo-Hyung Kim, Hyung-Jeong Yang, Guee-Sang Lee

**Affiliations:** Department of Artificial Intelligence Convergence, Chonnam National University, 77 Yongbong-ro, Gwangju 500-757, Korea; phantrandacthinh2382@gmail.com (T.-D.-T.P.); shkim@jnu.ac.kr (S.-H.K.); hjyang@jnu.ac.kr (H.-J.Y.)

**Keywords:** emotion recognition, electroencephalogram, EEG signals, channel correlation, frequency band correlation, multiscale kernel

## Abstract

Besides facial or gesture-based emotion recognition, Electroencephalogram (EEG) data have been drawing attention thanks to their capability in countering the effect of deceptive external expressions of humans, like faces or speeches. Emotion recognition based on EEG signals heavily relies on the features and their delineation, which requires the selection of feature categories converted from the raw signals and types of expressions that could display the intrinsic properties of an individual signal or a group of them. Moreover, the correlation or interaction among channels and frequency bands also contain crucial information for emotional state prediction, and it is commonly disregarded in conventional approaches. Therefore, in our method, the correlation between 32 channels and frequency bands were put into use to enhance the emotion prediction performance. The extracted features chosen from the time domain were arranged into feature-homogeneous matrices, with their positions following the corresponding electrodes placed on the scalp. Based on this 3D representation of EEG signals, the model must have the ability to learn the local and global patterns that describe the short and long-range relations of EEG channels, along with the embedded features. To deal with this problem, we proposed the 2D CNN with different kernel-size of convolutional layers assembled into a convolution block, combining features that were distributed in small and large regions. Ten-fold cross validation was conducted on the DEAP dataset to prove the effectiveness of our approach. We achieved the average accuracies of 98.27% and 98.36% for arousal and valence binary classification, respectively.

## 1. Introduction

Affective computing is one of the major research topics of advanced human-computer interaction for enhancing the experiences of virtual reality, video game interaction, educational system, and mental disorders detection. A wide range of modalities were carefully analyzed for the insight of mental state information and their applications to emotion recognition. In the early stages of this field, external data, such as facial expression [1], speech [2] and gesture [3], were favored because they were interpretable and tangible cues fitting the humans’ general knowledge, and the data was ubiquitous and could be collected on large scale in the laboratory environment or in the wild. Despite these advantages, the external data has a defect as humans, on some occasions, have the tendency to conceal their feelings, and their expressions may not correspond with the actual emotion. Due to the requirement for more reliable types of data, internal information such as brain signal, heart rate, blood pressure, and skin conductance response [4], that are hard to counterfeit, have recently been appealing to the work of affective computing. However, there were some drawbacks to the broad deployment of this approach. The measurement instruments for internal signals used to be bulky and complicated to manage. Nowadays, they are handy, and some types of data could be aggregated by wearable devices, such as watches. Among many kinds of physiological signals, electroencephalogram (EEG) signals, which record the neural activities, have been drawing the most attention thanks to its reliability and objectivity. Through electrode pads placed on the scalp, EEG devices could noninvasively measure the brain waves that reflect the many aspects of the conditions of the brain, including mental illness, sleep disorders, concentration level, and emotional state. EEG-based emotion recognition research is still captivating, since the approaches for feature extraction (time domain, frequency domain, electrode position, etc.) and the classification models (spatial, temporal) are considerably diversified.

The first challenge in emotion recognition using brain signals is the variation among individual neural activities within the same elicitation or emotional reaction. In other words, directly transferring the nervous system-related knowledge of one subject to another usually leads to incompatibility or poor generalization. To get the highest accuracy of emotion prediction out of a certain subject, a subject-dependent approach, which collects and learns only from the emotional data of that person, is preferred. However, this is not practical because individual calibration is time and data consuming. It is necessary to build a subject-independent model where the prior insights from a large number of subjects could apply to a new one. Hardly do all extracted features bring positive effects to the ability of emotion recognition of the new subject. The architecture of the model needs a feature selection mechanism so as to acknowledge the domain-invariant features that are useful on both training and testing data. Hence, in our method, we determine the transferable features from the beginning, which is one of several approaches to impede on the deficiency in subject generalization.

A conventional EEG-based emotion recognition system consists of four main stages, namely preprocessing, feature extraction, feature selection, and classification [5]. Feature extraction plays an important role in the success of emotion recognition. Brain signals can be probed in different domains (time, frequency, time-frequency, etc.) and from each domain, there are many corresponding features. Time-related features can be named as fractal dimension feature [6], Hjorth feature [7], Principal Component Analysis [8], high order crossing feature [9], statistical feature [10] (mean, standard deviation, variance, and first and second difference), etc. To get the frequency domain features, the raw EEG signals in the time domain are firstly converted by Fast Fourier transform or autoregressive [11] algorithms into five main bands: Delta (1–4 Hz), Theta (4–8 Hz), Alpha (8–14 Hz), Beta (14–31 Hz), and Gamma (31–50 Hz). From the frequency bands, the common extracted features are power spectral density, differential entropy [12], differential asymmetry, and rational asymmetry [13]. For the time-frequency domain features, the same procedure as the frequency domain was applied, but the algorithm for signal conversion was wavelet transform [14] or wave packet decomposition [15]. After obtaining the desired features, classifiers based on machine learning were proposed to discern the feature patterns into emotions. Support vector machine [16] and its variants [17] are the most used classifiers in papers. Another effective classifier is k-nearest neighbor, which was employed in [18]. With the rapid development of deep learning (DL) techniques, the components of the aforementioned system may be partially or entirely replaced by DL modules. Large numbers of network architectures that are based on convolutional neural networks (CNNs) [19] and recurrent neural networks (RNNs) [20] were proposed. The performance dominance of DL-based methods over the earlier ones are undeniable and they open new ways to process and analyze the EEG signals. Brain signals can be converted into another domain and represented as one, two, or three dimensional matrices depending on the attributes that were beneficial for authors’ ideas. Kwon et al. [21] designed a 2D CNN to learn the spectrogram image from the wavelet transformation of all the channels. To mine the temporal characteristics, Yan et al. [22] applied the long short-term memory (LSTM) network on one dimensional segmentations of raw signals under different temporal scales and concluded that smaller time scale shows better recognition performance. Salama et al. [23] focused on spatiotemporal feature learning and investigated the capability of 3D CNN on valence and arousal prediction. The utilization of machine learning or deep learning-based methods was diversified based on what characteristics of EEG signals the authors consider the most meaningful. They have not had the common agreement on the option of EEG feature types or classifier algorithms for emotion recognition.

The second problem is that most of the research about EEG-based emotion recognition collect the best features manually from several domains or automatically from the deep learning models but overlook the interactions among multi channels and multi bands. The conventional features and models are merely the reflection of neural activities on individual channels, while connectivity patterns or the activities of brain regions do have the impact on the emotional state [24]. Furthermore, higher frequency bands, such as Beta and Gamma, are more relevant to emotion recognition than the two lower bands [25], and the combination of four bands together could bring up the inter-band information that complements each other [26]. There have been several pieces of research that made their models aware of this information. The EEG data could be expressed in a specialized way that classification models were able to perceive the connectivity patterns among channels [23,27], or the models was built with certain mechanisms that could accumulated this information by themselves [28,29]. Motivated by this issue, in the proposed method, we first select the EEG features from the time domain in order to ensure the good generalization on the testing dataset and, at the same time, transform the extracted features into a 3D matrix that could delineate the channel and frequency band correlations. Noticing the differences in correlation between one channel and other close and remote channels, a multi-scale kernel CNN was constructed to capture the spatial information in short and long-range dependencies. The proposed network not only learns the emotional pattern of 32 channels of EEG signals, but also considers the interactions between four frequency bands. The DEAP dataset [30] was utilized to evaluate the performance of our approach on ten-fold cross validation, which is a subject-independent validation scheme. The proposed model achieves better classification performance than the model that used a filter with one size only and other recent methods about EEG-based emotion recognition.

The contributions of our paper are described as follows:

- We construct the network with multi-scale kernel convolution blocks to learn the local and global patterns of the preprocessed EEG features. The network could learn the salient connectivity patterns that have variation in structure.
- Utilizing the channel and band interaction information is effective for emotion recognition based on EEG signals. The time domain-based features along with the correlation information clearly enhance the performance of this task.

The remainder of this paper is organized as follows. Section 2 reviews the previous works on EEG-based emotion recognition. In Section 3, the materials are introduced and the details of our work are discussed. Section 4 describes the experimental schemes and explains the results achieved by the proposed method. We discuss and conclude our work in Section 5.

## 2. Related Works

The methods for EEG-based emotion recognition are varied in the choices of features and classifiers. Some of the papers extracted the features manually before forwarding to the next procedure while others built networks that could automatically process raw EEG signals and predict the emotion in sequence. Zheng [31] proposed the group sparse canonical correlation analysis method for emotion recognition and channel prediction. He concluded that the selected channels performed competitively compared to when all of the channels were utilized. Rozgic et al. [32] segmented the EEG signals into multiple overlapping segments and features were extracted separately from each segment. After that, they calculated the probability vectors of all segments that belonged to the same classes and forwarded them to the SVM with radial basis kernel. Zhuang et al. [33] employed a two-part unsupervised model to learn the rich and compact segment-level features. For a better representation of EEG signals, before extracting signal features, Pandey et al. [34] applied variational mode decomposition to raw signals and then passed them through a deep neural network. To integrate the feature learning from spatial and temporal information, Zhang et al. [35] employed a multidirectional recurrent neural network that scanned the temporal sequences and the spatial slices with different directions. Chen et al. [36] used Fisher score and Davies-Bouldin index to select helpful features from four physiological modalities, and the hidden Markov model as the classifier. Reference [37] used a probabilistic neural network as the classifier for the power features of four EEG frequency bands and also chose the most important channels by the ReliefF algorithm. Kwon et al. [21] extracted the spectrogram from EEG signals and the rate of signal change from galvanic skin response signal. Then, a fusion CNN model was applied to both extracted features to predict the emotional state. Kim et al. [38] integrated an attention mechanism into the LSTM model to pick up the part of the raw time-series that had the most influence on the emotion prediction. Besides learning the spatial and temporal features of the raw EEG-signals, attention-based convolutional recurrent neural network [39] with the channel-wise attention mechanism was introduced to figure out the channels that were more related to emotions and improve the average accuracy by approximately 30% compared to the CNN-RNN without using it.

Apart from the conventional feature selection from multi domains, several papers also regarded the correlations among EEG channels. To reduce the complexity of emotion recognition, Cheng et al. [27] employed the deep forest on the 2D frame sequence constructed from raw values, in order to learn the spatial position relationship. 3D CNN was favored by Salama et al. [23] to recognize emotions from multichannel EEG signals. Yin et al. [28] combined the graph CNN and LSTM to generate the graph domain features and memorize the change of relationship between two channels. Reference [25] captured the spatial information among channels by a hierarchical CNN. Song et al. [29] proposed the dynamical graph CNN to learn the relationships among various EEG channels in a dynamic way, which could convert the connections into discriminative features for emotion prediction. To better transfer the knowledge from the source domain to target domain, Wang et al. [40] utilized the symmetrical and positive definite matrix as the main feature set and its properties on the Riemannian manifold as a way to boost the generalization. In [41], LSTM modules acquired the features from 32 channels and the extracted features from each channel went through an attention mechanism to select emotion-related channels. Furthermore, a domain discriminator as the second branch was attached to make the distributions between the training and test domain similar. In our method, we proposed a 2D CNN with multiple kernel size at each convolution layer. The proposed model not only learns the position relationship among channels but also acquires the interaction between four frequency bands from the 3D feature representation of EEG signals.

## 3. Proposed Method

### 3.1. Dataset and Preprocessing

We adopt the publicly available dataset DEAP [30] to conduct the experiments and verify the effectiveness of our method. This dataset provides peripheral physiological and EEG signals of 32 subjects with an equal number of male and female participants. They were asked to watch 40 one-minute music videos as the sources of emotion elicitation, and during that time, 32 electrodes placed on the scalp according to the international 10–20 system recorded the activities of neural signals. Each subject had 40 trials corresponding to 40 videos, and there was a total of 1280 signal sequences (32 subjects × 40 trials) in the dataset. The first 3 s when participants had not watched the movies were used as baseline signals and the next 60 s were used for emotion recognition. After watching each clip, subjects were asked to rate the emotional state as valence, arousal, dominance, and liking in the range of 1 to 9. Valence and arousal are dimensional expression of basic emotions that we usually encounter. For instance, high arousal and positive valence relates to emotions, such as delight and happiness, while low arousal and negative valence discloses tiredness or depression. DEAP dataset prepares the raw version and the preprocessed version of the recorded EEG signals; for simplicity, we used the latter version that was downsampled to 128 Hz and filtered by a band-pass filter with cut-off frequencies of 4 and 45 in order to cut out electrooculogram artifacts and noises.

**Figure 1:**
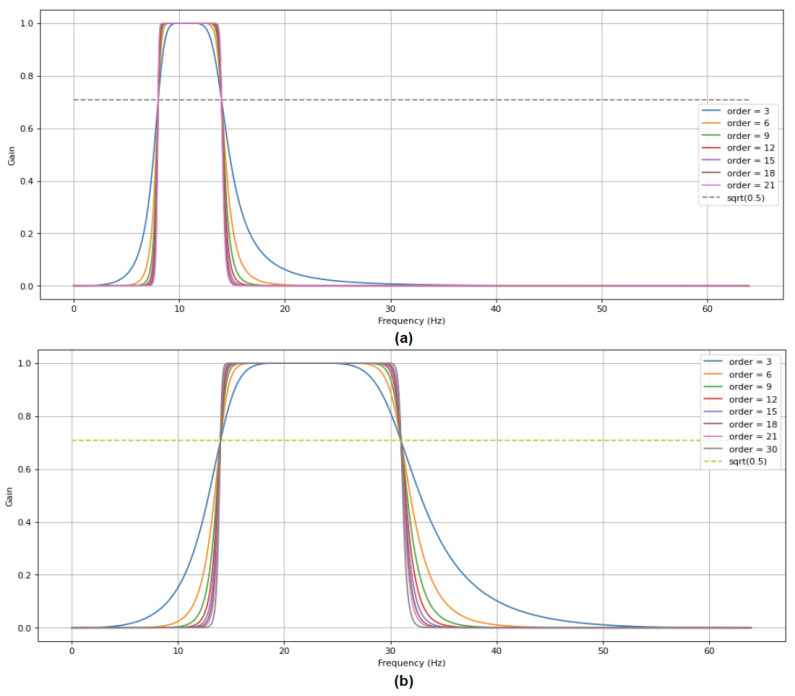
The effect of different orders of Butterworth band-pass filter (**a**) on Alpha frequency band and (**b**) on Beta frequency band.

According to the proposed method, the preprocessed signals were decomposed by a Butterworth filter into four frequency bands, namely Theta band, Alpha band, Beta band, and Gamma band. The Delta band was not concerned because the preprocessed signals no longer involved the frequency data from 1 to 4 Hz. The signals in the frequency domain after being filtered by the band-pass filters were transformed back to the time signals for further analysis. The high cut and low cut frequencies of Theta, Alpha, Beta, and Gamma bands were (4, 8), (8, 14), (14, 31), and (31, 50), respectively. As shown in Figure 1, the choice of band-pass filter’s parameter such as order value is important. To ensure that we could extremely minimize the area of the transition band and acutely attenuate the stop band, the order of the Butterworth filter was chosen as 30. Hence, the overlap between adjacent bands after filtering is trivial. From the time signal of each frequency band, the corresponding signals were divided into n segments through a sliding window in the main 60 s section (we choose 10 as the value of n in our method). The durations of segments were identical, and there was no overlapping between two adjacent segments on both training and testing datasets. The segments were considered as independent samples and acquired the labels of the trials from which they are derived.

For emotion analysis, we only use the self-assessment levels of arousal and valence states. Three types of classifications were carried out, namely low/high arousal, low/high valence, and high arousal high valence/high arousal low valence/low arousal high valence/low arousal low valence. The split point for low and high indices was 5.

### 3.2. Multi Feature Extraction and Feature Representation

**Figure 2:**
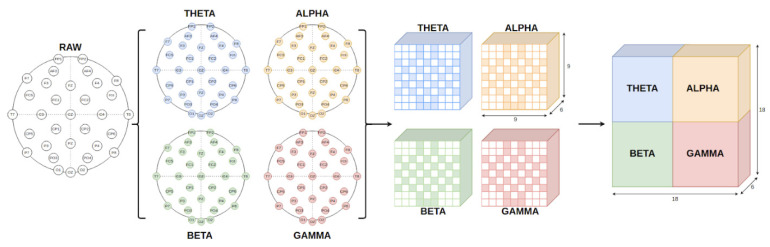
The framework of the feature extraction procedure.

The overall procedure of the feature extraction framework is shown in Figure 2. Based on the experiments, the time-domain features were preferred to represent the characteristics of EEG signals. Time-related features provide the most discriminative feature space out of several domains we have examined, and subsequently, we improve the emotion classification according to this foundation. The extracted features include differential entropy (DE), mean ($\bar{µ}$), mean of the first difference ($\bar{{µ}_{1}}$), mean of the second difference ($\bar{{µ}_{2}}$), variance ($\sigma^{2}$), and standard deviation (σ). Those features require low computational cost and few steps to acquire. The equations for each feature were described as follows:

(1) $$\mathrm{DE}\left(X\right)=-\int_{-\infty }^{+\infty }p\left(x\right)\log\left(p\left(x\right)\right)\mathrm{dx},$$

where X is the sequence of continuous random variables in a segment, and p(x) is the probability density function of segment X.

(2) $$\bar{\mu }\;=\frac{1}{n}\;\left(\overset{n}{\underset{i=1}{\sum }}x_{i}\right),$$

(3) $$\bar{\mu_{1}}=\frac{1}{n-1}\;\left(\overset{n-1}{\underset{i=1}{\sum }}(x_{i+1}-x_{i})\right),$$

(4) $$\bar{\mu_{2}}=\frac{1}{n-2}\;\left(\overset{n-2}{\underset{i=1}{\sum }}\left(x_{i+2}-x_{i}\right)\right),$$

(5) $$\sigma =\sqrt{\frac{1}{n}\;\left(\overset{n}{\underset{i=1}{\sum }}{\left(x_{i}-\;\bar{\mu }\right)}^{2}\right),}$$

(6) $$\sigma^{2}=\frac{1}{n}\;\left(\overset{n}{\underset{i=1}{\sum }}{\left(x_{i}-\;\bar{\mu }\right)}^{2}\right).$$

After calculating the features of all of the segments, they need to be normalized since the magnitudes of features could be extremely small or large. This could harm the learning process of the classification model when the variation is too large. We consecutively normalized a batch of samples within the same trial. The equation is shown as follows:

(7) $$f_{i}=\frac{f_{i}-{\;f}_{\min}}{f_{\max}-{\;f}_{\min}}$$

where f_i_ is the value of feature of one segment, f_min_ is the minimum value of all features of the segments from one trial, and f_max_ is the maximum value of all features of the segments from one trial.

Emotional states from neural signals could be derived not only from the changes of individual channels, but also from the interactions among them and frequency bands. To exploit the spatial relationship of electrodes, we need to compress this information into the input of our network to regulate its learning process, or the proposed network should be constructed with the ability to self-analyze and figure out this information by some specialized mechanisms. For our method, we choose the former one. The features from 32 electrodes were mapped into a 2D matrix with the size of 9 × 9 for each frequency band. The positions of electrodes still follow the international 10–20 system of EEG electrode scalp placement, since retaining position information is convinced to be more efficient in learning the relations among channels. The mapping matrix is depicted in Figure 2. The 32 values were placed in designated positions, while the rest of the positions were filled with value zeros. There were four matrices based on this structure to represent the four frequency bands. They were treated as different modalities of EEG signals. We combine them as one larger 2D matrix as the early fusion approach so the classification can explore the interaction of both channels and frequency bands. We do not stack them as channels of images because we reserve the third dimension for multi-feature representation. One matrix expresses one type of feature only. Therefore, six matrices were constructed to display all the extracted information. The size of one sample was 18 × 18 × 6, in which the width and the height had the same proportion, and the channel length was 6. The 3D feature matrix could simultaneously manifest the relationships among distinct channels and frequency bands and provide the feature mixture from the time domain.

### 3.3. Deep Learning Model for Emotion Recognition

**Figure 3:**
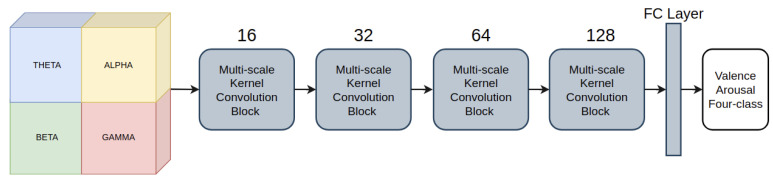
The proposed method.

CNN-based methods, specifically 2D CNN, were adopted to have the full benefit of the predetermined EEG structure. The 2D convolution models could detect complex edges and shape-related features more efficiently than traditional or machine learning methods. The neural network is self-designed to fulfill our requirements and be compatible with the input matrix. The architecture of our proposed model is illustrated in Figure 3. Our model has four convolution blocks that share the same structure and one fully connected layer. The numbers of output channels for each convolution block are 16, 32, 64, and 128, respectively.

**Figure 4:**
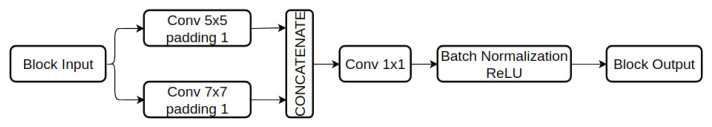
The architecture of each multi-scale kernel convolution block in the network.

**Figure 5:**
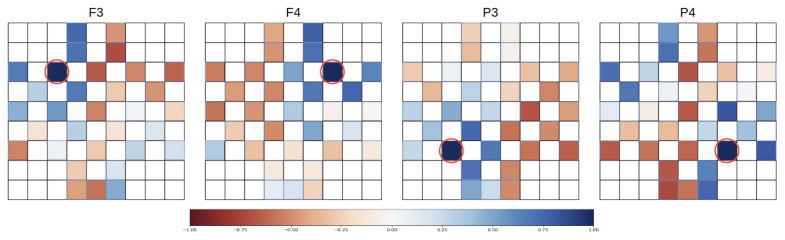
The Pearson correlation map between selected channels and the others. High positive correlation is presented as the dark blue color, and high negative correlation is presented as the dark red color. White means there is no correlation. The selected channels (F3, F4, P3, and P4) are marked with red circles.

As the size of the input feature matrix was quite small and the pooling layer could result in the loss of crucial information, this layer is not present in our model. Figure 4 depicts the architecture of the proposed convolution block. Each block contains two convolution layers at the beginning with distinct kernel sizes, in particular 5 × 5 and 7 × 7. Large kernel sizes were suitable for 2D feature representation, because each electrode commonly had high correlation of neural activities with adjacent electrodes, but the relationships among signals from different brain regions were manifold. They may be positive, negative, or even no correlation found between a couple of electrodes. Figure 5 demonstrates the correlation properties of the selected channels against the others. We used Pearson correlation to evaluate the interconnection among channels. Four correlation images were taken from one trial of a random subject.

From the Figure 5, the designated channel usually has high correlation with the adjacent channels, which means the information in those are likely to be similar. Hence, we need to enlarge the feature region to acquire wide range of data from remote pairs of channels. Moreover, the input of the network was the combination of four matrices from four frequency bands. Small filter size causes the lack of diverse information about the features and the connectivity patterns. In the proposed block, large kernel size was utilized to capture the global patterns, and smaller kernel size was expected to study the local patterns. The two convolution layers received the same input and after passing it through them, we obtained two kinds of output features, from small to large, and the outputs of the two layers had the same size. Subsequently, the two outputs were concatenated, and a convolution layer with kernel size of 1x1 reduced the dimension of the combined features. A 1 × 1 convolution can not only decrease the dimensions, but also cut down the number of parameters and improve computational efficiency. Batch normalization and ReLU layers come after to complete the block.

## 4. Experiments and Results

### 4.1. Experiemental Setup

We conduct the experiments with ten-fold cross validation, which is a subject independent validation scheme. All the trials from 32 subjects were selected and 10 non-overlapping segments were derived from each trial. The length of each segment was 6 s. The total number of samples for evaluation was 12,800 (32 subjects × 40 trials × 10 segments). We randomly split all of the samples into 10 sets and a ten-fold cross validation scheme was utilized to evaluate the performance. After 10 iterations, the final result was the average accuracy of ten folds.

Classification accuracy was used to evaluate all the experiments in our paper. For arousal or valence classification, we use binary cross-entropy loss, because the labels of them are high value and low value. In four emotion classification, categorical cross-entropy was utilized as the main loss. The learning rate was initially set as 0.001, and the cosine annealing method as learning rate scheduler gradually decreased the learning rate until it reached one tenth of the beginning learning rate at the last epoch. Cosine annealing helped the model to converge better and avoided the large variations of performance on the testing dataset. The total number of epochs was 150 and the batch size was set as 640. Adam algorithm was used as the optimizer for our neural network. The proposed model was designed and built with Python environment and Pytorch framework.

### 4.2. Results

At first, the features from four different domains were examined to find the most compatible type of features for our network to implement the emotion recognition task based on EEG features. The time-domain features were mentioned earlier in Section 3. For frequency-domain features, power spectrum density and differential entropy were selected to represent this domain. To get the features from the time-frequency domain, we convert the raw signals from the time domain into this domain by wavelet transform algorithm. Mean, standard deviation, variance, and entropy were extracted from the time-frequency domain to evaluate the performance. Correlations among channels were also considered. We used Pearson correlation and phase locking value as indices for this domain. Pearson correlation describes what degree and direction of one continuous signal was proportional to the other signals in linear correlation. Pearson index ranges from −1 to +1. Negative one means the two data were inversely proportional, and vice versa, while zero means there is no correlation between the two data. Phase locking value is a measure of phase synchrony between two time-series data and the unit of it is radian or angle degree. We extract the features from raw signals only to grade the performance of each domain, which means the information from four frequency bands was not considered here. Hence, the input size of the image in Table 1 is 9 × 9 × k. The width and the height have the same size of 9 pixels, and k is the number of features depending on each domain (for instance, k is 6 in time domain and 4 in time-frequency domain). The overall performance is shown in Table 1. Time domain features achieve the best results, which are 89.92%, 89.24%, and 84.48% on arousal, valence, and mixed classification, respectively. It also leads the time-frequency features by a margin of nearly 10 percent of accuracy on arousal and valence classification tasks. Frequency domain-based features obtained an accuracy of 54.91% on four-class classification, while correlation domain-based features got the worst performance, with an accuracy of only 41.84%. Three types of features with lower scores performed well on the training set, but the acquired parameters did not generalize well on the testing dataset, and it appeared to be overfitting. Features from the time domain have high transferability, behaving reliably on both training and testing dataset.

In the second experiment, we examined the effectiveness of each frequency band, and the combination of them on the emotion recognition. As illustrated on Table 2, Gamma band, and Beta band highly relate to emotional state, while Theta band and Alpha band had lower impact on the task of emotion prediction. The achieved results were consistent with the conclusion of the research by [25]. Despite the high performance, the utilization of only one band cannot surpass the utilization of raw signal. The emotional states could be better inferred by exploring the interactions of four frequency bands. The combination of two of the best frequency bands and all of them were put into the test in order to prove the benefit of incorporated information. The fused representation of them before inputting these into the model conforms with what we presented in the previous section. The Beta-Gamma combination achieved the accuracy of 94.28%, 94%, and 93.05% of accuracy on arousal, valence, and four-class classification, respectively, which were greater than those of raw signal. The results indicate that the incorporated features from the top bands complement each other to generate higher accuracy than individual band’s features. Furthermore, the combination of four frequency bands improved the prediction accuracy significantly on three classification tasks by approximate 4% of accuracy. The superior performance of the full combination of four frequency bands’ features proves that the interdependence of them could interpret more comprehensively the neural activities related to emotions.

Based on the aforementioned representation of the extracted features from EEG signals, the proposed model must be able to learn not only the local patterns, such as frontal and parietal brain regions, but also the global patterns, like the correlation between two hemispheres or among four frequency bands. In Table 3, specific kernel sizes and their associations were investigated to find the best way to learn multi-scale features. Among three individual kernel sizes, the model constructed with only kernel size 3 × 3 obtained the worst accuracies. This result is explainable, because the electrodes next to each other tend to behave similarly and so too their extracted features, which could not contribute extensive information for the target task. The accuracies of emotion prediction were proportional to the kernel size. The long-distance relation was more valuable to the structure of our model. In terms of combination, the mixture of kernel sizes 5 × 5 and 7 × 7 produced the best result on four-class classification, which was 98.38% accuracy. The performances of valence and arousal classification of the last three rows in Table 3 were slightly similar. The achieved results consolidate our idea of employing multiple feature sizes so that the neural network could capture the local and global patterns, enhancing the capability of emotion recognition.

In Table 4, we compare the arousal and valence binary classification to other papers with the same validation scheme. All the results are produced on the DEAP dataset. Our method achieves better results than the other methods in Table 5. Cheng et al. [36] also employed the spatial position relationship between 32 channels, and they used cascade forest structure to scan through the matrix with a 3 × 3 sliding window. The size of the sliding window may be too small to cover a larger area so that the information is related to local patterns only. Kim et al. [35] used the LSTM model to automatically extract features from 32 channels of raw EEG signals, and an attention layer to determine which channel features had influence on the output. Our network with the ability to exploit the interactions among channels and four frequency bands achieve the accuracies of 98.27% and 98.36% on arousal and valence classification, respectively, which proves the effectiveness of the proposed method.

### 4.3. Ablation Study

To prove that the results of the analysis do not depend on where the signal was cut, we did extensive experiments on the 5 × 5 and 7 × 7 multi-scale kernel model (the one that got the best result in emotion recognition) in Table 5. The first row was for the current results of the model. The second row was when we choose the starting point of the first segment of each trial at one second after the end of the baseline signal (originally, we chose the starting point right after the end of the baseline signal). The third row showed the results when we chose it at two seconds after the end of the baseline signal, and we follow that setup in the next rows. From the table, we can see that the results do not change considerably when we change the starting point of the signal.

## 5. Conclusions

In this paper, we prove that the time-domain based features were the best type of features for our approach, and the adoption of channel and band correlation properties improved the performance of EEG-based emotion recognition. To take advantage of this awareness, we proposed the multi-scale kernel CNN to learn the EEG feature patterns with variation of size. The proposed model combined the local information and global information of channels to study the neural activities among electrodes that were related to emotional states. The experimental results on the DEAP dataset ascertain the effectiveness of our method.

## Figures and Tables

**Table 1:** Classification accuracies on features from different domains.

Domain	Arousal	Valence	Four-Class
Time	89.92	89.24	84.48
Frequency	68.77	68.05	54.91
Time–Frequency	79.59	80.05	69.84
Correlation	65.27	60.88	41.84

**Table 2:** Classification accuracies on each frequency band and their combination.

Frequency Bands	Arousal	Valence	Four-Class
Theta	74.10	71.74	59.19
Alpha	74.72	71.71	58.94
Beta	84.52	84.70	76.25
Gamma	87.39	86.91	81.10
Raw	89.92	89.24	84.48
Beta-Gamma	94.28	94.00	93.04
All	98.27	98.36	98.38

**Table 3:** Classification accuracies of the proposed network on different kernel sizes.

Kernel Size	Arousal	Valence	Four-Class
3 × 3	88.82	87.07	84.12
5 × 5	94.45	93.75	93.30
7 × 7	97.23	97.36	97.31
3 × 3 + 5 × 5	96.63	96.16	96.38
3 × 3 + 7 × 7	98.38	98.15	98.12
5 × 5 + 7 × 7	98.26	98.36	98.38
3 × 3 + 5 × 5 + 7 × 7	98.29	98.38	98.12

**Table 4:** Comparison among previous research on arousal and valence binary classification.

Kernel Size	Year	Arousal	Valence
Kwon et al. [21]	2018	76.56	80.46
Zhang et al. [37]	2016	81.21	81.76
Yin et al. [28]	2021	85.27	84.81
Salama et al. [23]	2018	88.49	87.44
Kim et al. [38]	2020	88.30	90.10
Cheng et al. [27]	2021	97.53	97.69
The proposed method	2021	98.27	98.36

**Table 5:** Classification results on different starting points of the signals.

	Arousal	Valence	Four-Class
Shift 0 s	98.26	98.36	98.38
Shift 1 s	98.94	98.72	98.23
Shift 2 s	98.37	98.28	97.80
Shift 3 s	98.23	98.30	97.97
Shift 4 s	98.34	98.25	97.88
Shift 5 s	98.40	98.26	97.93

## Data Availability

Not applicable.
